# Associations of microbial and indoleamine-2,3-dioxygenase-derived tryptophan metabolites with immune activation in healthy adults

**DOI:** 10.3389/fimmu.2022.917966

**Published:** 2022-09-29

**Authors:** Niknaz Riazati, Mary E. Kable, John W. Newman, Yuriko Adkins, Tammy Freytag, Xiaowen Jiang, Charles B. Stephensen

**Affiliations:** ^1^ Graduate group of Molecular, Cellular, and Integrative Physiology, University of California, Davis, Davis, CA, United States; ^2^ USDA Western Human Nutrition Research Center, University of California, Davis, Davis, CA, United States; ^3^ Department of Nutrition, University of California, Davis, Davis, CA, United States; ^4^ West Coast Metabolomics Center, Genome Center, University of California, Davis, Davis, CA, United States

**Keywords:** tryptophan, inflammation, immunity, indole, indole acetic acid (IAA), indole propionic acid (IPA), indole producing bacteria, kynurenine

## Abstract

**Background:**

Tryptophan (Trp) metabolites from intestinal bacteria (indole, indole acetic acid [IAA] and indole propionic acid [IPA]), and the Trp metabolite kynurenine (Kyn) from the indoleamine 2,3-dioxygenase (IDO) pathway, are aryl hydrocarbon receptor (AhR) agonists and thus, can regulate immune activity *via* the AhR pathway. We hypothesized that plasma concentrations of these metabolites would be associated with markers of immune activation in a cohort of healthy adults in a manner consistent with AhR-mediated immune-regulation. We also hypothesized that the plasma Kyn/Trp ratio, a marker of IDO activity, would be associated with immune markers reflecting IDO activation in innate immune cells. Finally, we hypothesized that some intestinal bacteria would be associated with plasma indole, IPA and IAA, and that these bacteria themselves would be associated with immune markers.

**Methods:**

A novel set of 88 immune markers, and plasma Trp metabolites, were measured in 362 healthy adults. Bacterial taxa from stool were identified by 16S rRNA gene analysis. Multiple linear regression analysis was used to identify significant associations with immune markers.

**Results:**

The sum of indole and IAA was positively associated with natural killer T-cells levels. Kyn and Kyn/Trp were positively associated with neopterin and IP-10, markers of type 1 immunity, and TNF-α and C-reactive protein (CRP), markers of the acute phase response, and the regulatory cytokine IL-10. Three bacteria negatively associated with Trp metabolites were associated with markers of immune activation: the family *Lachnospiraceae *with higher lymphocyte counts but lower level of activated CD4 T-cells, the genus *Dorea* with higher production of IFN-γ by T-cells in PBMC cultures, and the genus *Ruminococcus* with higher production IL-6 in PBMC cultures stimulated with bacterial lipopolysaccharide (LPS).

**Conclusions:**

In this cohort of healthy adults bacterial Trp metabolites were not strongly associated with immune markers. Conversely, the Kyn/Trp ratio was strongly associated with markers of systemic inflammation and the acute phase response, consistent with IDO activation in innate immune cells. Finally, commensal bacteria associated with lower plasma (and perhaps intestinal) levels of bacterial Trp metabolites were associated with greater immune activation, possibly reflecting decreased regulatory immune activity related to lower intestinal levels of bacterial indole metabolites.

## Introduction

Tryptophan (Trp) is an essential amino acid with an estimated dietary requirement of 3.5 to 6.0 mg/kg of body weight per day for adults. Dietary protein digested in the small intestine is the primary source of Trp. Trp is efficiently absorbed through the small intestine and enters the blood circulation where it is either bound to albumin, or present in the free form. The free form of Trp is primarily used in the body for protein synthesis, but it is also used for synthesis of serotonin and Kynurenine (Kyn). Trp is converted to Kyn by either tryptophan 2,3-dioxygenase (TDO) mainly expressed in the liver, or indoleamine 2,3-dioxygenase (IDO-1/IDO-2) expressed in the intestinal epithelium and many other types of cells including immune cells (1–3). TDO expression is induced by stress hormones such as cortisol and Trp while IDO expression is induced by inflammatory mediators, such as interferon (IFN)-α, IFN-γ, TNF-α, and by microbial components, such as lipopolysaccharide (LPS) and lipoteichoic acid through the activation of TLRs (4–7). IDO-dependent Trp metabolism in the immune cells is activated in response to inflammation. The plasma Kyn/Trp ratio is often used to reflect IDO activity in immune cells (3, 8).

Small amounts of dietary Trp not absorbed in the small intestine, as well as Trp from exfoliated intestinal epithelial cells, proteins secreted into the intestinal lumen, and bacterial proteins, reach the colon and can be metabolized by commensal bacteria. In the colon, Trp may be metabolized by intestinal bacteria to indole and its derivatives such as indole acetic acid (IAA) and indole propionic acid (IPA). Also, high levels of microbiota-derived indole may contribute to retrograde Trp synthesis from indole, providing another source of Trp for microbial metabolism (9). Indole and other microbially derived Trp metabolites are absorbed through the intestinal epithelium and enter the blood circulation.

Some metabolites of Trp including Kyn, indole, IAA and IPA are ligands of the aryl hydrocarbon receptor (AhR), a transcription factor in many immune cells involved in modulation of immune activity (10–13). Currently, diet-derived AhR ligands are hypothesized to have health-promoting effects *via* regulation of the immune system (12, 14). The Trp metabolites indole, IAA, IPA and Kyn are found in the blood of healthy adults at levels consistent with biological activity (9, 15, 16). Previous studies have examined the association of plasma Trp metabolite concentrations in extreme situations, such as between groups with and without disease and/or in association with a limited number of immune markers (2, 5, 7, 17). The current study sought to extend these investigations by evaluating plasma Trp metabolite associations with a novel set of 88 immune biomarkers in a large cohort of healthy adults. While these individuals are healthy, some are at an increased risk of immune activation due to common risk factors for inflammatory disease, including age (2) and obesity (18).

Thus, the primary goal of this discovery analysis is to determine if the Trp-derived AhR agonists Kyn, indole, IAA and IPA are associated with immune function markers of innate and adaptive immunity in a manner consistent with the currently proposed protective, immune modulatory role of such diet-derived AhR agonists in a cohort of healthy U.S. adults using a novel set of immune biomarkers. In parallel, we have also examined the association of the Kyn/Trp ratio as a marker of IDO activity to account for the likely association of IDO activity with markers of systemic immune activation, as would be expected from the literature (8). A second goal of our study is to identify intestinal microbiota associated with plasma indole, IAA and IPA concentrations, and to determine if these taxa are themselves associated with markers of systemic inflammation and immune activation.

## Methods

### Study design

Healthy adults were recruited into the Nutritional Phenotyping Study conducted at the USDA Western Human Nutrition Research Center (*ClinicalTrials.gov*: NCT02367287) as previously described (19, 20). Recruitment targets in this observational study were balanced by sex, three age categories (18-34, 35-49, 50-66 y) and three BMI categories (18-24, 25-29, 30-44 kg/m^2^). Data on dietary intake and physical fitness were collected during study visits and fasting blood was collected to measure Trp metabolites and immune function. For the current analysis, data were available from 362 study volunteers. Ethical approval for this study was received from the Institutional Review Board of the University of California, Davis, as previously described (19, 20).

### Dietary intake

Habitual dietary intake was assessed using the Block 2014 Food Frequency Questionnaire (FFQ) by NutritionQuest (21). Proximal dietary intake of individuals was also assessed from 24-hour recalls collected by the Automated Self-Administered 24-Hour (ASA24) Dietary Assessment tool (22).

### Physical fitness

Physical fitness was assessed using the YMCA Step Test (23) after excluding volunteers with risk factors that would preclude this sort of physical activity (e.g., back or knee problems) using a physical activity readiness questionnaire from the American College of Sports Medicine (24).

### Blood collection

Blood was drawn in the morning after a 12 h overnight fast (water being allowed to maintain hydration) following consumption of a standard meal the evening before (19).

### Isolation of peripheral blood mononuclear cells

PBMC were isolated from blood (using sodium heparin as an anticoagulant) using a single-step density gradient prepared with Ficoll-Histopaque 1077 (Sigma Aldrich, St. Louis MO, USA).

### CMV antibody test

Previous studies reported that cytomegalovirus (CMV) seropositive and seronegative individuals differ in levels of CD4 and CD8 T-cell subsets, possibly due to repeated reactivation of CMV infection expanding memory and effector/memory T-cell subsets (25, 26). To adjust for this potential difference in our analysis of the correlation of immune biomarkers with Trp metabolites, we assessed infection with CMV by measuring antibody status using fasting sodium heparin plasma analyzed with the CMV IgG Reagent Pack with analysis run on the Roche Diagnostics (Indianapolis, IN) Cobas e411 autoanalyzer according to the manufacturer’s instructions. Results are categorized as positive (“reactive”), borderline, and negative (“not-reactive”). One sample in this study was borderline and was considered negative during statistical analysis.

### PBMC culture methods

Freshly isolated PBMC were washed using Hank’s Balanced Salt Solution (Thermo Fisher scientific, Pleasanton Ca, USA) and resuspended in Russ-10 medium. Russ-10 was prepared using a 500 mL bottle of RPMI 1640 (Thermo Fisher Scientific) with the addition of 5 mL

L-Glutamine (Thermo Fisher Scientific, “L-Gluta MAX”), 5 mL of antibiotic/antimycotic (Thermo Fisher Scientific), 5 mL non-essential amino acid mixture (Thermo Fisher Scientific), 5 mL Sodium Pyruvate (100 nM; Thermo Fisher Scientific), 5 mL HEPES (4-(2-hydroxyethyl)-1-piperazineethanesulfonic acid) buffer (Thermo Fisher Scientific), and 0.25 ml of 100 mM beta-Mercaptoethanol (Sigma Aldrich). Russ-10 was used in cell culture containing 10% heat-inactivated fetal bovine serum (Thermo Fisher Scientific). PBMC were cultured with 5% CO2 at 37° C in a humidified incubator at a final concentration of 1 x 10^6^ cells/mL for 24 h with LPS stimulation, or for 48 h with T-cell-specific stimulation in 96-well flat-bottom polystyrene plates (Corning, Corning PA). Supernatants were collected by centrifugation at 300 x g for 10 min at 4°C and immediately frozen at -80°C until analysis of cytokine concentrations. LPS (from *E. coli* 0111: B4; List Biologicals, Campbell Ca USA) was included at a final concentration of 5 ng/mL, a dose that was determined to be sub-maximal in preliminary experiments with PBMC from healthy adults (data not shown). An equal volume of endotoxin-free water (Sigma Aldrich) was used as a negative control for LPS in parallel cultures. T-cell stimulation was provided by anti-CD3 plus anti-CD28 monoclonal antibodies (Thermo Fisher Scientific, anti-human CD3 mouse isotype IgG2a, and anti-human CD28 isotype IgG1). Isotype control antibodies (Thermo Fisher Scientific; mouse IgG1 and mouse IgG2a) were used as the negative control. Antibodies were diluted to 3 μg/mL (individual concentration) in Phosphate Buffered Saline (PBS; Thermo Fisher Scientific) and used to pre-coat plates (50 μL/well) at 4°C overnight using anti-CD3 plus anti-CD28 monoclonal antibodies for T-cell stimulation and the two isotype controls together as the negative control.

### Measuring cytokine concentrations from PBMC cultures

PBMC culture supernatants were analyzed for cytokine concentrations using the MSD U-plex Platform, U-plex Biomarker group 1 (human) Multiplex Assay using a U-plex 5-assay 96 well sector plate (MESO Scale Discovery, Rockville MD USA) with the MSD sector imager 2400 (MESO Scale Discovery) or the Mesoscale SQ 120. PBMC cultures stimulated with anti-CD3 plus anti-CD28 antibodies, or with the corresponding isotype control were diluted 1:10 and 1:2, respectively, for determination of interferon-γ and interleukin (IL)-10, IL-13 and IL-17A concentrations. PBMC cultures stimulated with LPS, or its negative control, were diluted 1:40 and 1:2, respectively, for determination of tumor necrosis-factor (TNF)-α, IL-1β, IL-6 and IL-10 concentrations. Mean concentrations (μg/L) of duplicate wells were used for analysis.

### Complete blood count with differential

During this four-year recruitment period (June 2015 through July 2019), the CBC analyses were performed using whole blood (treated with EDTA as an anticoagulant) in the UC Davis Health, Department of Pathology and Laboratory Medicine Clinical Laboratory using a Beckman Coulter LH750/780 (prior to October 2016) or a Beckman Coulter DxH800 automated hematology analyzer, with the exception that twelve samples early in the study (prior to August 14, 2015) were analyzed on an Abbott Cell-Dyn 322 analyzer at the WHNRC.

### Plasma immune markers measured by ELISA in fasting plasma

Neopterin concentration (nmol/L) was measured using undiluted sodium heparin plasma using a commercial, competitive enzyme immunoassay (Alpco, BRAHMS GmbH, Salem, NH, USA) according to the manufacturer’s instructions. Myeloperoxidase concentrations (μg/L) were measured using sodium heparin plasma (1:10 dilution) using a commercial ELISA kit (Alpco Immunodiagnostic, Salem, NH, USA) according to the manufacturer’s instructions. Soluble CD14 (sCD14) concentrations (μg/L) were measured in duplicate using sodium heparin plasma (1:400 or 1:600 dilution) using a commercial ELISA kit (Bio-Techne R&D Duoset Systems, Minneapolis, MN USA) according to the manufacturer’s instructions. Plates for these three assays were read on an Agilent BioTek Synergy reader (Santa Clara, CA USA) and data analyzed using the BioTek Gen5 software.

### Plasma immune markers measured by multiplexed assay in fasting plasma

The concentrations of 16 plasma proteins (μg/L) were measured in plasma using MSD assay kits and the MSD sector imager 2400 (MESO Scale Discovery). EDTA plasma was used for 13 proteins (CRP, SAA, ICAM-1, VCAM-1 using the Vplex Vascular Injury Panel 1 with samples diluted 1:1,000; eotaxin, IP-10, MCP-1 and MDC using the Vplex Custom Human Biomarker Chemokine Panel 1 with samples diluted 1:4; TNF-α, IL-1β, IL-6, IL-8 and IL-10 using the Vplex Custom Human Biomarker Proinflammatory Panel 1 with samples diluted 1:2) and sodium heparin plasma for 3 proteins (MMP1, MMP-3 and MMP-9 using the MMP 3-plex Ultrasensitive Kit with samples diluted 1:10). Three levels of lyophilized controls were used on each plate to assess plate-to-plate variation. Mean concentrations (μg/L) of duplicate wells were used for analysis.

### Flow cytometry of fasting peripheral blood

Four flow cytometry panels were used for analysis of leukocytes using both isolated PBMC (panels A-C) and whole blood (panel D). PBMC were resuspended in a final volume of 50 μL of Brilliant Stain Buffer (BD Biosciences, San Jose, CA, USA) with one million PBMC for each panel. Then PBMC were stained with the Fixable Viability stain 510 (BD Biosciences) on ice prior to staining with the rest of the antibodies. For panel D, after staining 100 μL whole blood with antibodies, erythrocytes were lysed with BD FACS Lysing solution, and the cell pellet was washed and resuspended in staining Buffer (BD Biosciences, San Jose, Ca, USA) before analyzing sample on BD LSR Fortessa flow cytometry. Antibodies (**Table S5**) and gating strategies (**Figures S1 – S4**) for these four panels are shown in supplemental materials: Panel A - naïve and central/effector memory T-cells, with activation markers CD38 and HLA-DR; Panel B - Th1, Th2, Th17 Cells, NK Cells and B Cells; Panel C - total and memory Treg cells with activation markers CD38 and HLA-DR; and Panel D - total T-cells plus monocytes (classic, intermediate and alternative), neutrophils, and eosinophils with activation marker CD11b. Cells were analyzed using an LSRFortessa flow cytometer (BD Biosciences) configured with blue (488nm), red (640nm), violet (405 nm) and UV lasers (355nm). Data were collected using FACSDiva and analyzed using FlowJo version 10.6.1 software (BD Biosciences).

### Plasma tryptophan metabolites

Plasma Trp metabolites were measured using MxP^®^ Quant 500 kits (Biocrates Life Sciences AG, Innsbruck, Austria) in the presence of deuterated internal standards as per manufacturer’s instructions. Fasting plasma from 362 participants were randomized onto five plates. A single subaliquot of NIST Standard Reference Material 1950: Metabolites in Human Plasma (Sigma-Aldrich, St Louis, MO) was processed on each plate along with method blanks, and manufacturer provided quality control plasma dilutions and 7pt calibration curves for a subset of metabolites including Trp and Kyn. Trp and Kyn results were corrected by recoveries of deuterated analogs of the respective compounds, while indole, IAA, and IPA were quantified against single point calibrations relative to various deuterated acylcarnitines. All reported metabolites were detected with negative mode ionization on an API 6500 QTRAP (Sciex, Framingham, MA) after chromatographic separations. Data was processed with manual curation of peak integrations using MultiQuant v 3.2 (Sciex). Peak areas were imported into MetIDQ Oxygen (Biocrates Life Sciences AG). Results were corrected for plate specific effects by normalization to the median of four mid-range quality control solutions analyzed on each plate. For the replicate NIST plasma, the coefficients of variation for Trp, Kyn, IAA and IPA across the 5 analyzed plates were 9%, 13%, 40% and 20%, respectively. Indole was not detected in this sample.

### Bacterial 16S rRNA gene sequence analysis

Amplification and sequencing of the 16S rRNA V4 – V5 region from bacterial DNA extracted from stool was performed by the Dalhousie University Integrated Microbiome Resource using primers 515F, GTGYCAGCMGCCGCGGTAA, and 926R, CCGYCAATTYMTTTRAGTTT (27–29) as previously described (30). Sequences were analyzed using Qiime2 version 2019.10 (31) also as previously described (30).

### Statistical analyses

Statistical analyses were conducted using SAS 9.4 (SAS Institute, Cary, North Carolina, United States). Correlation analysis between dietary Trp/protein intake and Trp metabolites was calculated using the Spearman test, and a p-value<0.05 was considered statistically significant. Group comparisons of Trp metabolites across age and BMI categories and sex were performed by the nonparametric Kruskal-Wallis test and differences between groups were compared using the Dwass–Steel–Critchlow–Fligner (DSCF) multiple comparison adjustments.

Association of Trp metabolites with the 88 immune biomarkers were characterized using a multiple linear regression model with Trp metabolites as the independent variables and individual groups of immune biomarkers as the dependent variables. Variables were all normalized using rank-based normal transformation prior to analysis. The analysis was controlled for age and BMI groups, and sex. It was also controlled for CMV infection status as CMV infection history confirmed by the reactive IgG level was associated with some immune markers in our study, primarily but not exclusively differences in T-cell measures (data not shown). Spearman correlation analysis between Trp metabolites and YMCA step scores showed that IPA had a significant positive association with the YMCA step scores (data not shown). For this reason, correlation analysis between IPA and the immune variables was also controlled for the YMCA step scores. To account for multiple comparisons, Benjamini-Hochberg adjusted p-values (32) were computed within six groups of immune biomarkers including A. effector/memory T-cells and activation levels (n=24), B. other lymphocytes including Th cells, NK, NK T-cells and B cells (n=11), C. PBMC cytokines(n=8), D. complete blood count (n=15), E. innate cell activation (n=11), and F. plasma markers (n=19). Statistical significance was set at p< 0.05. Heatmaps were plotted to observe the correlation between Trp metabolites and immune markers. Beta values of the standardized regression coefficients estimated by the linear regression, and the corresponding raw and adjusted p-values are documented in **Supplemental Table S3**. The standardized regression coefficients were used to represent effect size (33). The size of the standardized effects used conventionally are 0.1-0.3 for small effect, 0.3-0.5 for moderate effect, and 0.5-1 for large effect (34, 35).

For the association of Trp metabolites and microbial taxa, the likelihood ratio test in the DESeq2 package (v 1.26.0) (36) from R (v 3.6.3) (37) was used to identify differentially abundant taxa at the family and genus levels between three groups of rank-normalized Trp metabolites. Significant Trp metabolites-microbiota associations were determined based on Benjamini-Hochberg adjusted p-values of<0.05.

Associations of microbial taxa with immune variables were determined using a linear regression analysis performed in SAS. Each model included sex, age and BMI categories, and CMV infection status as covariates, the significant Trp metabolites-associated taxa as the independent variables, and individual groups of immune biomarkers as the dependent variables. Significant associations were determined based on Benjamini-Hochberg adjusted p-values of<0.05. All p-values are documented in the corresponding **Supplemental Tables**.

## Results

### Description of study volunteers

Study participants were from the WHNRC Nutritional Phenotyping Study which is a cross-sectional observational trial and included healthy adults, men and women, aged 18–66 y, with BMI (kg/m^2^) 18–44, and living near Davis, California. The recruitment strategy and basic demographic characteristics of the participants have been described previously (20). Participant characteristics by sex, age, and BMI categories for those included in the present analysis are summarized in **Table 1**.

**Table 1:** Characteristics of healthy adult participants who provided fasting blood samples.

	Sex		Age, y			BMI (kg/m^2^)			Participants, n	% Total
**Sex**	Men	Women	18-34	35-49	50-66	18-24	25-29	30-44	362	100
Men	172	–	60	61	51	67	67	38	172	47.5
Women	–	190	69	58	63	72	64	54	190	52.5
**Age, y**										
18-34	60	69	129	–	–	50	48	31	129	35.6
35-49	61	58	–	119	–	48	35	36	119	32.9
50-66	51	63	–	–	114	41	48	25	114	31.5
**BMI (kg/m^2^)**										
18-24	67	72	50	48	41	139	–	–	139	38.4
25-29	67	64	48	35	48	–	131	–	131	36.2
30-44	38	54	31	36	25	–	–	92	92	25.4

### Description of plasma metabolites by sex, age, and BMI categories

**Figure 1:**
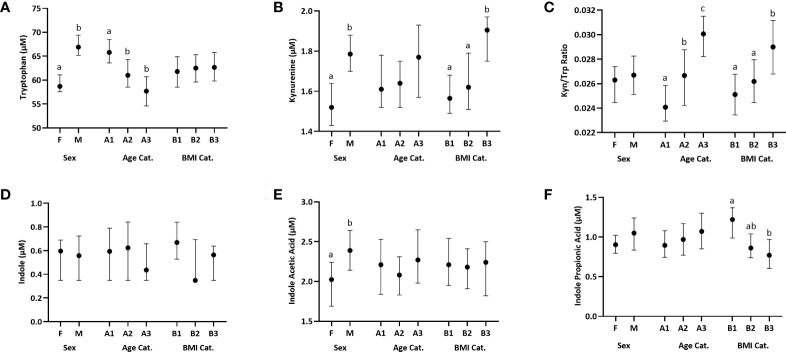
Distribution of plasma Trp and Trp metabolites across females (F) and males (M), three age categories (Cat.), A1-A3: [18–34 y (n=129), 35–49 y (n=119), and 50–66 y (n=114)] and three BMI categories, B1-B3: [18–24.99 kg/m^2^ (n=139), 25.00–29.99 kg/m^2^ (n=131), and 30.00–44 kg/m^2^ (n=92)]. Data are presented as medians, and 5th and 95th percentiles. Significant differences between groups within the sex, age and BMI categories are marked by different letters. **(A)** Trp, **(B)** Kynurenine, **(C)** Kynurenine : Tryptophan (Kyn/Trp) ratio, **(D)** Indole, **(E)** Indole Acetic Acid, **(F)** Indole Propionic Acid.

Sex, age, and BMI can affect immune function. We thus examined plasma Trp metabolite concentrations using these three categorical variables that were used to balance recruitment by sex, age, and BMI categories prior to examining associations with immune variables. Our study population includes 172 men and 190 women. Compared to women, men had a significantly higher concentration of Trp (p<0.0001), Kyn (p=0.0002) and IAA (p= 0.015) (**Figures 1A, B, E**; **Table S1C**). The three age groups were A1:18–34 y (n=129), A2: 35–49 y (n=119), and A3:

50–66 y (n=114). The youngest age group (group 1) had a significantly higher Trp concentration compared to each of the two older groups (group 1 vs 2: p=0.0006, group 1 vs 3: p=0.0001) (**Figure 1A**; **Table S1A**). The Kyn/Trp ratio was significantly different across the three age groups, with the ratio increasing with age (group 1 vs 2: p =0.004, group 1 vs 3: p= 0.0001, group 2 vs 3: p=0.034) (**Figure 1C**; **Table S1A**). The three BMI groups were B1:18–24.99 kg/m^2^ (n=139), B2: 25.00–29.99 kg/m^2^(n=131), and B3: 30.00–44 kg/m^2^ (n=92). The group with the lowest BMI, group 1, had a significantly higher IPA concentration compared to the group with the highest BMI, group 3 (p=0.0002) (**Figure 1F**; **Table S1B**). Kyn concentration was significantly higher in group 3 vs group 1 (p=0.005) and group 2 (p=0.024) (**Figure 1B**; **Table S1B**). Similarly, Kyn/Trp ratio was significantly higher in group 3 vs group 1 (p=0.005) and group 2 (p=0.009) (**Figure 1C**; **Table S1B**). Indole concentrations did not differ by sex, age or BMI (**Figure 1D**; **Table S1**).

### Association of dietary tryptophan and protein intake with plasma metabolites of tryptophan

To determine if habitual dietary Trp intake was associated with Trp metabolite concentrations in plasma, we examined the relationship between Trp and total protein intake from FFQs, estimating cohort usual intake over the preceding year, and protein intake from 24-hour recalls collected by the ASA24 by Spearman analysis. These analyses showed that FFQ-based dietary Trp and protein intake, as well as ASA24-based protein intake, were positively associated with plasma Trp (FFQ Trp intake: p=0.003; FFQ protein intake: p=0.002, ASA24 protein intake: p=3.3E-07*) and Kyn (FFQ Trp and protein intake: p=0.02, ASA24 protein intake: p=0.04) concentrations. FFQ- and ASA24-based protein intake were also positively associated with IAA (FFQ protein intake: p=0.04, ASA24 protein intake: p=0.01) (**Table S2**).

### Association of plasma tryptophan metabolites with markers of inflammation and immune activation

We used multiple linear regression analysis to examine the relationships between plasma Trp metabolites and 88 immune variables in six categories (**Table S6**): (A) effector and memory T-cell subsets and their level of activation; (B) other lymphocytes including Th1, Th2, Th17, natural killer T (NKT), NK, and B cells; (C) cytokines produced by PBMC stimulated with LPS or with anti-CD3 plus anti-CD28 antibodies to activate T-cells; (D) standard complete blood count with differential; (E) innate cells types and activation level of these types, and; and (F) plasma cytokines, chemokines, acute phase proteins, matrix metalloproteinases and other markers of immune activation.

**Figure 2:**
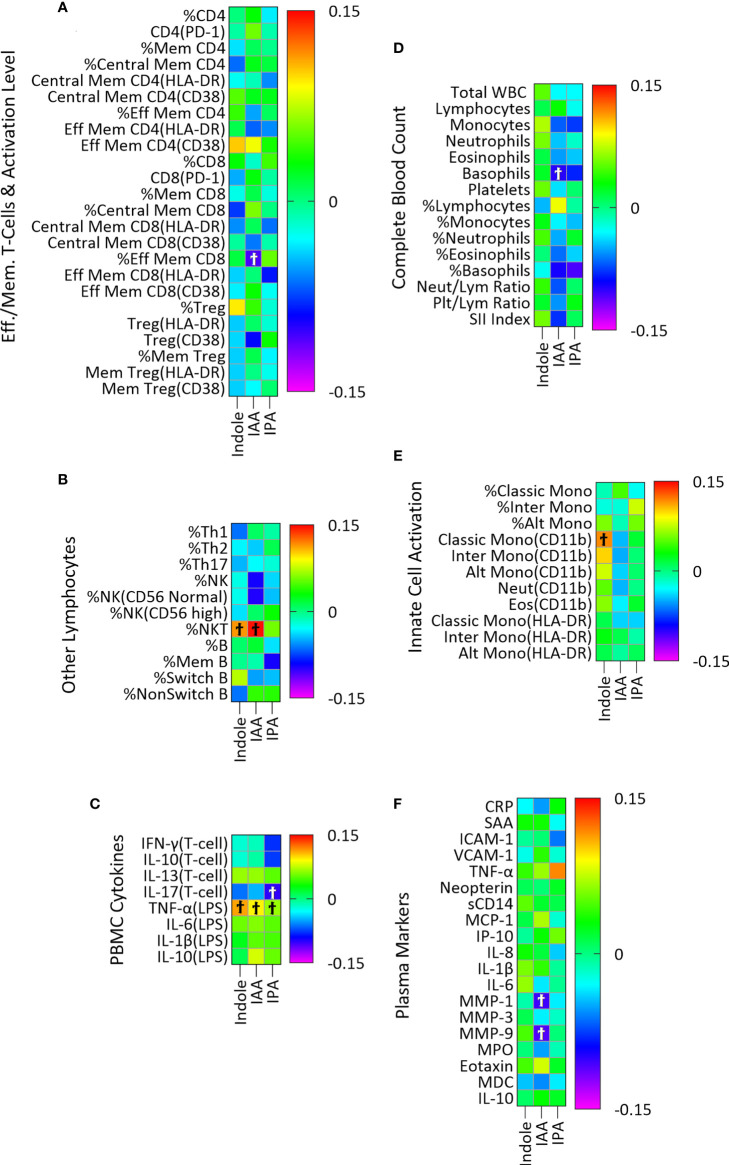
Association of plasma indole, IAA and IPA with plasma markers of immune activity in a linear regression model adjusted for age, sex, and BMI categories and CMV infection status. The association of IPA with immune markers was also adjusted for YMCA step scores. Heatmaps **(A–F)** show coefficients of association with adjusted beta values for the six immune groupings **(A)** Effector/Memory T-cells and activation level (n = 24), **(B)** Other lymphocytes including Th cells, NK, NK T-cells and B cells (n=11), **(C)** PBMC cytokines (n=8), **(D)** Complete Blood Count (n = 15), **(E)** Innate cell activation (n=11), and **(F)** Plasma (n = 19). ‘Ϯ’ indicates significant with unadjusted p values <0.05. No significant associations were seen using with Benjamini-Hochberg adjusted p-values <0.05.

The microbial metabolites of Trp included in this analysis were indole, IAA and IPA. Plasma indole was significantly associated with three, IAA with six, and IPA with two immune variables though none of these associations remained significant after adjustment for multiple comparisons (**Figure 2**; **Table S3C**).

**Figure 3:**
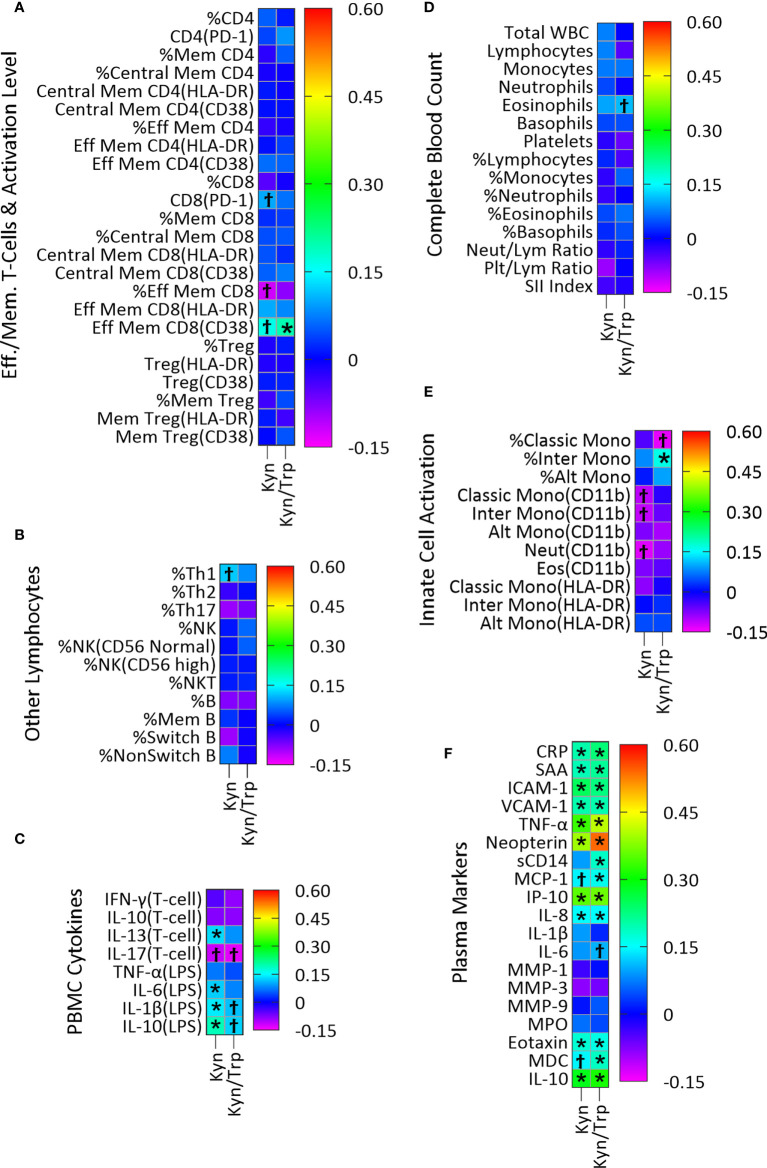
Association of plasma Kyn and Kyn/Trp with plasma markers of immune activity in a linear regression model adjusted for age, sex and BMI categories and CMV infection status. Heatmaps **(A–F)** show coefficients of association with adjusted beta values for the six immune groupings **(A)** Effector/Memory T-cells and activation level (n=24), **(B)** Other lymphocytes including Th cells, NK, NK T-cells and B cells (n=11), **(C)** PBMC cytokines (n=8), **(D)** Complete Blood Count (n=15), **(E)** Innate cell activation (n=11), and **(F)** Plasma (n=19). ‘Ϯ’ indicates significant association with unadjusted p values<0.05. ‘*’ indicates significant association with Benjamini-Hochberg adjusted p-values<0.05. **(A)** PD-1, Programmed Death-1; Mem, Memory; Eff, Effector; **(B)** NK, Natural killer cells, NKT, Natural killer T-cells; **(D)** Neut, Neutrophils; Lym, Lymphocytes; Plt, Platelets; SII, (Platelet×Neutrophil)/Lymphocyte; **(E)** Mono, Monocytes; Alt, Alternate; Inter, Intermediate; Eos, Eosinophils; **(F)** CRP, C-Reactive Protein; SAA, Serum Amyloid A; ICAM-1/VCAM-1, Intracellular/Vascular cell adhesion molecule; TNF-α, Tumor Necrosis Factor-α; sCD14, Soluble CD14; MMP, Matrix metalloproteinase; MPO, Myeloperoxidase; MDC, Macrophage-derived chemokines.

Plasma concentration of the host metabolite Kyn was significantly associated with twenty-four immune variables, fourteen of which remained significant after adjustment for multiple comparisons (**Figure 3**; **Table S3A**). In PBMC cultures, T-cell cytokine IL-13 and LPS-stimulated cytokines IL-6, IL-1β and IL-10 were positively associated with Kyn concentration (**Figure 3C**; **Table S3A**). Kyn was also positively associated with the plasma acute phase proteins CRP and SAA, the markers of vascular inflammation ICAM-1 and VCAM-1, the pro-inflammatory cytokine TNF-α, the regulatory cytokine IL-10, and the chemokines IL-8, eotaxin, and IP-10, and the marker of macrophage activation neopterin (**Figure 3F**; **Table S3A**).

Similarly, the Kyn/Trp ratio, included here as an index of IDO activity, was positively associated with twenty-one immune variables, fifteen of which remained significant after adjustment for multiple comparisons (**Figure 3**; **Table S3B**). Activated effector memory CD8 T-cells with CD38 activation marker (**Figure 3A**; **Table S3B**) and percentage of intermediate monocytes (**Figure 3E**; **Table S3B**) were positively associated with Kyn/Trp. Also, CRP, SAA, ICAM-1, VCAM-1, TNF-α, IL-10, the chemokines IL-8, IP-10, MCP-1, eotaxin, and MDC and the markers of macrophage activation neopterin and sCD14 were all positively associated with Kyn/Trp (**Figure 3F**; **Table S3B**).

**Figure 4:**
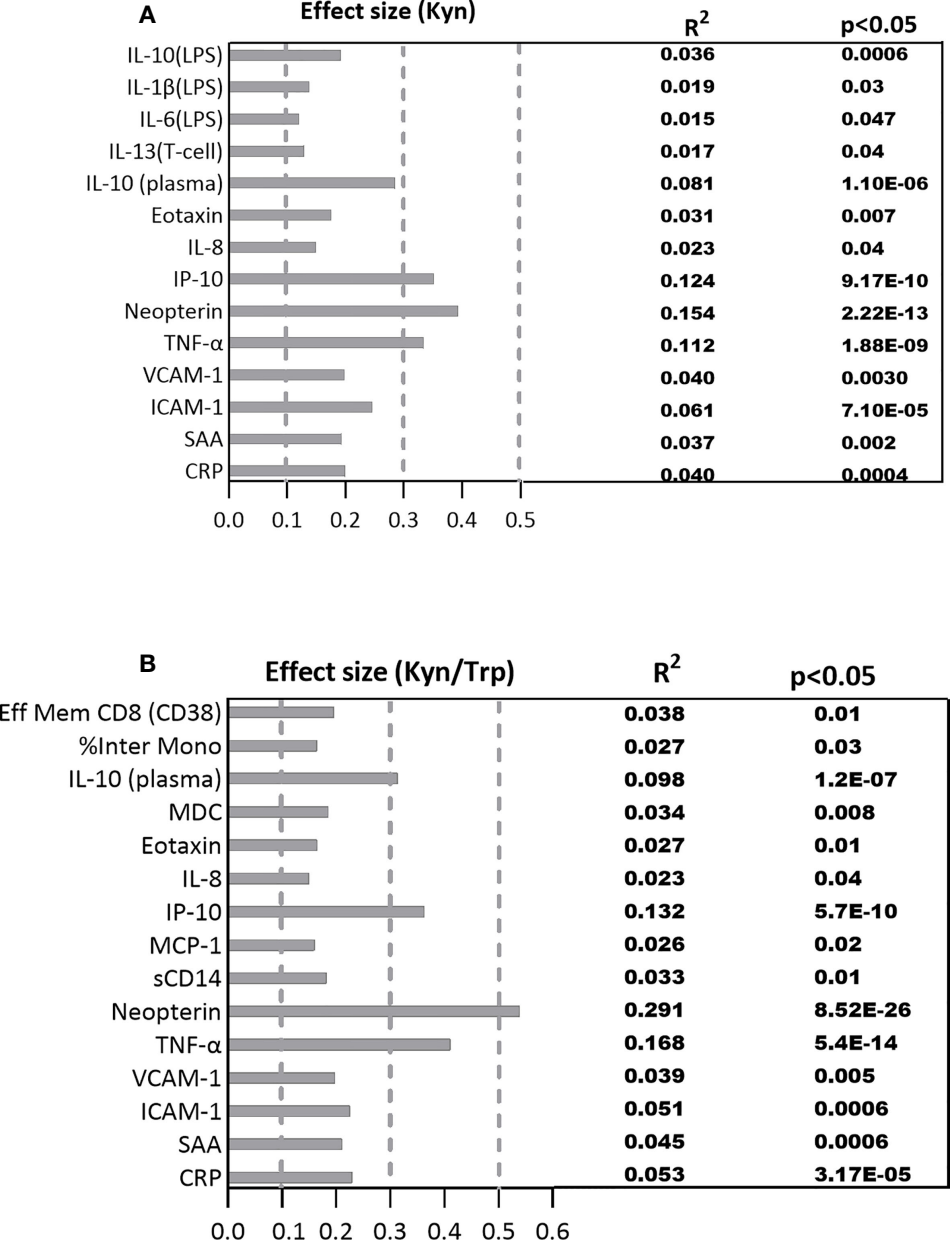
The standardized effect sizes, R², and p values for significant association between the immune biomarkers and **(A)** Kyn, **(B)** Kyn/Trp ratio. Dotted lines represent the range of the small (0.1-0.3), medium (0.3-0.5) and large (0.5-1) effects.

The estimated effect size of the significant correlations adjusted for multiple comparisons are represented in **Figures 4A, B**. The largest effect size among the significant correlations between plasma immune variables with Kyn falls within the medium range for the immune markers including TNF-α (*R* ^2^ =0.112, p=1.88E-09), IP-10 (*R* ^2^ =0.123, p=9.17E-10), and neopterin (*R* ^2^ =0.154, p=2.22E-13) which respectively account for 11.2%, 12.3%, and 15.4% of variations among the plasma immune variables. The effect size of the significant correlations between Kyn and the rest of the plasma immune markers and the PBMC cytokines is within the small range (**Figure 4A**).

The standardized effect size for significant correlations between plasma immune markers and Kyn/Trp falls within the medium range for the immune markers including IL-10 (*R* ^2^ =0.098, p=1.20E-07), IP-10 (*R* ^2^ =0.132, p=5.70E-10), and TNF-α (*R* ^2^ =0.168, p=5.40E-14) which account for 9.8%, 13.2%, 16.8% ofplasma Kyn and Kyn/Trp with plasma markers variations among the plasma immune markers while neopterin (*R* ^2^ =0.291, p=8.52E-26) with large effect size explains 29.1% of variation among the plasma immune markers. The effect size of the significant correlations between Kyn/Trp and the rest of the immune markers is within the small range (**Figure 4B**).

### Associations among tryptophan metabolites, gut microbiota, and immune markers

**Figure 5:**
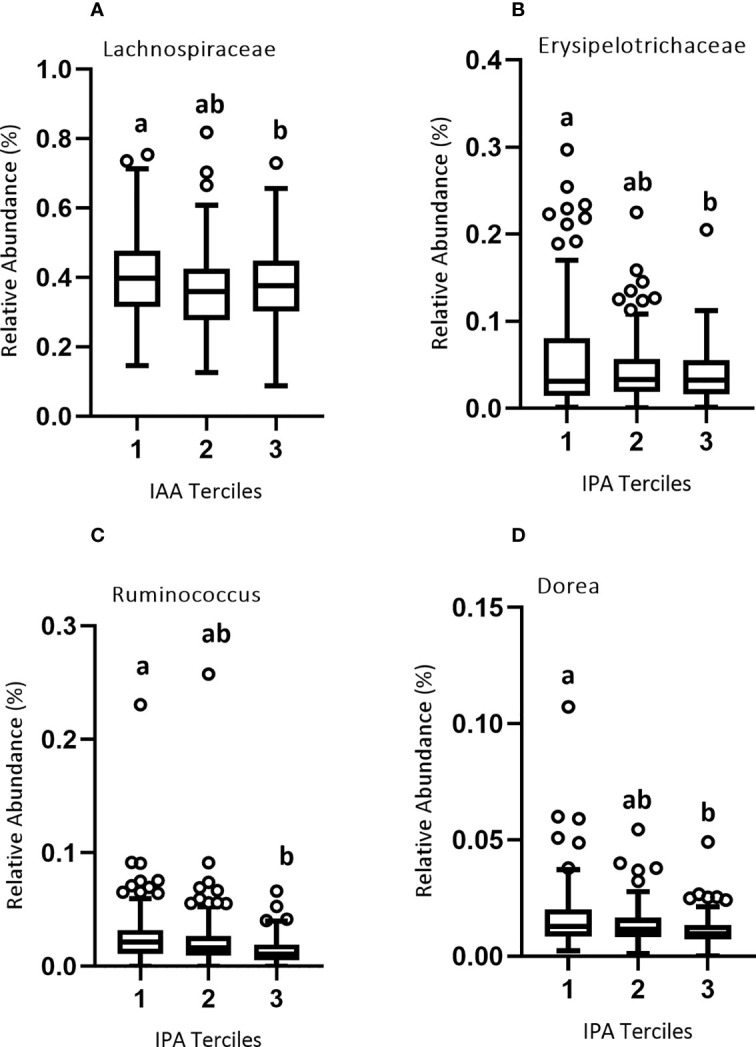
Relative abundance of microbial taxa across the three terciles of Trp metabolites. Variability of the percent relative abundance of microbial taxa across the three groups of Trp metabolites is presented using a Tukey's boxplot. The boxplot elements are defined as following: center line, median; box limits, upper and lower quartiles; whiskers, 1.5 × interquartile range. Circles indicate outliers. As described in Results, there was a significant negative association between **(A)** IAA and family Lachnospiraceae, and between **(B)** IPA and family Erysipelotrichaceae and **(C)** IPA and genera Ruminococcus and **(D)** Dorea. Significant differences between the groups of Trp metabolites are marked by different letters.

Since gut bacteria may metabolize Trp to produce bioactive metabolites, we conducted a microbiome-wide analysis to identify microbial taxa either positively or negatively associated with plasma Trp metabolite concentrations divided into lower, middle, and upper terciles 1-3 (**Figure 5**; **Table S4**). We reasoned that such taxa might themselves be associated with immune variables because their production (or catabolism) of Trp metabolites would help determine the plasma concentration of these metabolites. Our results were as follows: At the family level, there was a significant negative association between the relative abundance of *Lachnospiraceae* and IAA concentration of the lower and upper terciles 1 (0.002-1.58 μM) and 3 (2.79-11.4 μM) (**Figure 5A**), and between *Erysipelotrichaceae* and IPA concentration of the lower and upper terciles 1(0.001-0.653 μM) and 3 (1.37-23.3 μM) (**Figure 5B**). At the genus level, there was a significant negative association between the relative abundance of *Ruminococcus* and IPA of the lower and upper terciles (**Figure 5C**) and *Dorea* and IPA concentration of the lower and upper terciles (**Figure 5D**).

Next, we determined if these taxa were associated with markers of immune activation and inflammation using the same regression analysis approach as used for the metabolites themselves (**Table 2**). At the family level, relative abundance of *Lachnospiraceae* was significantly (p<0.05) positively associated with total lymphocyte concentration in peripheral blood and negatively associated with activated (CD38 positive) effector memory CD4 T-cells. At the genus level, relative abundance of *Dorea* was significantly positively associated with T-cell production of IFN-γ in PBMC cultures. Also, at the genus level, relative abundance of *Ruminococcus* was positively associated with LPS-stimulated IL-6 in PBMC cultures (**Table 2**).

**Table 2:** Association of Trp metabolite-associated microbial taxa with markers of immune activity.

Microbial Taxa	Immune Markers	β	SE	Raw p	Adjusted p
Genus *Dorea* (Family *Lachnospiraceae*)	**PBMC** (T-cell)-IFN-γ	0.158	0.047	0.0009	0.007*
	**Plasma**				
	MDC	-0.129	0.052	0.01	0.24
	MMP-3	0.081	0.041	0.05	0.86
	**CBC**				
	Monocytes	0.143	0.049	0.004	0.06
	Lymphocytes	0.099	0.049	0.05	0.54
	Platelet/Lymphocyte	-0.133	0.051	0.01	0.14
	**Flow Cytometry**				
	Central Mem CD4 T-cells (CD38)	-0.106	0.053	0.05	0.99
Genus *Ruminococcus*	**PBMC**				
(Family *Lachnospiraceae*)	(LPS)-TNF-α (LPS)-IL-6	0.097 0.128	0.046 0.045	0.04 0.005	0.22 0.04*
	(T-cell)-IL-13	0.124	0.047	0.008	0.06
	**CBC**				
	WBC	0.102	0.049	0.04	0.47
	Lymphocytes	0.125	0.049	0.01	0.18
	Monocytes	0.12	0.05	0.02	0.22
	Platelet/Lymphocyte	-0.125	0.052	0.02	0.22
Family *Lachnospiraceae*	**Flow Cytometry**				
	Treg (CD38)	0.117	0.054	0.03	0.68
	Central Mem CD4 T-cells (CD38)	-0.117	0.055	0.04	0.8
	Eff Mem CD4 T-cells (CD38)	-0.178	0.056	0.002	0.04*
	%Eff Mem CD8 T-cells	0.111	0.055	0.04	0.9
	%Memory B-cells	-0.151	0.055	0.006	0.06
	**Plasma**				
	MMP-9	0.124	0.055	0.02	0.45
	**CBC**				
	WBC	0.106	0.05	0.03	0.47
	Lymphocytes	0.153	0.05	0.002	0.03*
Family *Erysipelotrichaceae*	**CBC**				
	Eosinophils	0.125	0.051	0.02	0.21
	Lymphocytes	0.142	0.05	0.004	0.07
	**Flow Cytometry**				
	%Th2 cells	-0.123	0.059	0.04	0.41
	CD4 T-cells (PD-1)	-0.099	0.049	0.05	0.99
	**PBMC**				
	(T-cell)-IL-13	0.095	0.047	0.05	0.29
	**Plasma**				
	MMP-3	0.098	0.041	0.02	0.31
	MCP-1	-0.147	0.05	0.004	0.07

The table includes β (correlation coefficients), SE (standard error), unadjusted raw p-values<0.05 and Benjamini-Hochberg adjusted p-values. Asterisks indicate adjusted p-values<0.05.

## Discussion

### Association of dietary tryptophan intake with plasma tryptophan metabolites

Dietary total protein and Trp intake were both positively correlated with plasma Trp (**Table S2**), as would be expected for an essential amino acid (38). Also, they were both positively correlated with plasma Kyn (**Table S2**), the precursor of which is plasma Trp. Interestingly, total protein intake was also positively associated with plasma concentration of IAA, but not IPA or indole (**Table S2**) suggesting that dietary Trp intake may determine the plasma concentrations of some Trp metabolites produced by intestinal bacteria and thus potentially affect health *via* a microbiome-dependent pathway, in addition to the well-defined role of Trp in host metabolism.

### Tryptophan metabolism, age, and obesity

Plasma Trp concentrations were higher in men than women, as previously reported (39) and higher in the youngest category of study volunteers compared to the older two groups (**Figure 1A**). The reason for this age difference is uncertain but is not due to differences in protein or Trp intake as we saw no differences in dietary intake of total protein or specifically of tryptophan among these groups (data not shown). The positive association of Kyn/Trp with age (**Figure 1C**) is likely due to increasing IDO activity with age related to the phenomenon of inflammaging (2). Similarly, the higher Kyn and Kyn/Trp ratio in obese individuals compared to volunteers with a healthy BMI (**Figures 1B, C**) is presumably due to elevated IDO activity and inflammation in obesity (18). The lower IPA concentration in obese study subjects relative to normal-weight individuals (**Figure 1F**) is consistent with previous studies which also found a decreased plasma IPA level in obese individuals and reflects changes in the production of the gut microbial metabolites (40, 41).

### Association of tryptophan metabolites with immune activation and systemic inflammation

#### a. IDO-derived tryptophan metabolite Kynurenine and the Kyn/Trp ratio

Plasma Kyn, which can act as an AhR agonist (10, 11), was examined for possible novel associations with markers of immune activation in our study. However, since Kyn is produced as a result of IDO-1 activation by innate immune cells, most of the associations identified for Kyn concentration were also seen for the Kyn/Trp ratio, the index of IDO activity. The four associations seen for Kyn that were not identified as statistically significant for the Kyn/Trp ratio despite being similar in direction and magnitude (positive associations with *ex vivo* production of the cytokines IL-1β, IL-6, IL-10 and IL-13 by PBMC; **Figure 3C**) could represent novel associations not related to IDO-1 activity itself. As discussed below, IDO-1 activity in innate immune cells is associated with cytokine production and it is the likely cause of these associations, rather than AhR activation.

Many positive associations were seen for the Kyn/Trp ratio, primarily with plasma markers of systemic inflammation and the acute phase response, which likely represent associations caused by IDO activity from immune cells including dendritic cells and macrophages (42, 43). Consistent with this is the positive association of Kyn with LPS-induced IL-1β and IL-6 in PBMC supernatants (**Figure 3C**) indicating an association between IDO-1 activation and inflammatory cytokines which is reported by previous studies showing induction of IDO-1 by interferons in PBMCs and monocyte-derived macrophages (7, 44–47). Similarly, positive association of the Kyn/Trp ratio with percent of intermediate monocytes (**Figure 3E**) indicates association of monocytes with IDO activity and is consistent with previous studies which found that IDO-1 upregulation in monocytes/macrophages are associated with increased level of Kyn (6, 44).

With respect to plasma immune markers in **Figure 3F**, association of Kyn and Kyn/Trp with the macrophage activation marker neopterin has the highest effect size, and with TNF-α, the chemokine IFN-γ-induced protein-10 (IP-10), and IL-10 has the next highest effect sizes (**Figures 3A, B**). The robust positive association of Kyn and Kyn/Trp with neopterin and IP-10 (**Figure 3F**) were previously observed (47, 48) as were positive association of Kyn/Trp with MCP-1 and MDC, chemokines secreted by monocytes/macrophages and with sCD14, a marker of LPS-mediated activation of monocytes/macrophages (**Figure 3F**) indicate that increased IDO activity represented by the Kyn/Trp ratio is associated with immune activation and type 1 immunity.

Moreover, association of Kyn and Kyn/Trp with markers of inflammation in plasma including TNF-α, CRP, SAA, ICAM-1, VCAM-1, and IL-8 indicates that increased IDO activity represented by the Kyn/Trp ratio is associated with systemic inflammation and the acute phase response. The association of IDO activity with markers of type 1 immunity and markers of systemic inflammation and immune activation in healthy subjects of this study is consistent with the idea that the expression of IDO and production of Kyn, as well as these markers of systemic inflammation and immune activation, are all driven by a common underlying factor, such as obesity. Interestingly, there was also a positive association between Kyn and Kyn/Trp and plasma level of eotaxin, a chemokine associated with type 2 immunity, and IL-10, a key regulatory cytokine (**Figure 3F**). In addition, there was a positive association between Kyn and IL-13, a type 2 cytokine, and LPS-induced IL-10 production in PMBC cultures (**Figure 3C**). The concurrent positive association of Kyn with inflammatory cytokines and markers of type 1 immunity, as well as with mediators of type 2 and regulatory immunity indicate the broad association of the Kyn pathway with different types of immunity. It is also possible that the regulatory and type 2 immune associations result from regulatory response to the induction of type 1 immunity and systemic inflammation. Consistent with this suggestion are previous studies which show that Kyn and Kyn/Trp are positively associated with plasma neopterin and IL-10 concentrations in healthy adults (17) and that activation of IDO-1 by inflammatory cytokines, and Kyn production induces AhR activation in dendritic cells and macrophages which results in IL-10 secretion (49, 50).

#### b. Lack of association of microbial tryptophan metabolites with immune activation and systemic inflammation

We did not find significant association (after correction for multiple comparisons) between microbially derived Trp metabolites and the 88 markers of both innate and adaptive immune activation used in this study. There was a nearly significant positive association between IAA and percent NK T-cells (adjusted p = 0.06) and a non-significant association of similar magnitude (based on the beta coefficient) for indole (**Figure 2B**). When the sum of indole and IAA were used as an independent variable, the association with percent NK T-cells was significant (β=0.152, adjusted p=0.04) using the same regression model, suggesting a possible association with NK T-cell abundance. A previous study proposed that indole, through interaction with the transcription factor RORγt, and indole metabolites such as IAA, through interaction with AhR regulate the production of IL-17 and IL-22 by NK T-cells (51). The association of the sum of indole and IAA with NK T-cells observed in our study could indicate a combined effect of indole and IAA on regulation of NK T-cell cytokines. While these indole metabolites of Trp are known to regulate immune activity, as has been often described in the literature (9, 15, 16), the overall lack of association seen here is probably due to two principal factors. First, these metabolites are produced in the intestine and may act primarily on intestinal immune cells where concentrations are higher than in systemic circulation (52) and we may have seen different results had we been able to examine concentrations of these metabolites in intestinal tissues or stool samples. Second, we have studied a cohort of generally healthy adults. Though chronic inflammation is present in such individuals, particularly in older adults and those with obesity (as demonstrated by the association of IDO activity with age and BMI in this study), our sample size may be insufficient to identify associations of microbial indole metabolites with this relatively low level of immune activation. In addition to the association with NK T-cells, some weaker associations were identified (**Figure 2**) that may point to future directions for research.

### Association of gut microbiota with plasma tryptophan metabolites

Trp can be converted into indole, and its derivatives such as IAA and IPA, by intestinal bacteria such as *Bacteroides thetaiotaomicroin*, *Citrobacter* sp., *E. coli*, *Peptostreptococcus russellii*, *Clostridium sporogenes*, and *Lactobacillus* spp. (53–55). However, we did not find any positive correlation between gut bacteria and plasma levels of Trp metabolites derived from microbial metabolism (indole, IAA, IPA). One potential explanation for this lack of a positive association is that further metabolism of these compounds by host enzymes results in their lower concentration in plasma and obscures associations with intestinal bacteria. A study which reports that genetic factors which are responsible for further metabolism of IPA partially affect its circulating level supports this hypothesis (56).

Our study shows that the genera *Dorea* and *L-Ruminococcus*, both members of the family *Lachnospiraceae* were negatively associated with plasma IPA, and the family *Lachnospiraceae* itself was negatively associated with plasma IAA (**Figure 5**). Negative associations of *Lachnospiraceae* and *Ruminococcus* with plasma IPA have been previously reported (56) but the other associations, to our knowledge, have not been reported and their mechanism is not known. Further experiments would be needed to demonstrate a cause-effect association.

### Association of tryptophan metabolite-associated microbiota with immune activation and systemic inflammation

The family *Lachnospiraceae*, as well as the genera *Dorea* and *L-Ruminococcus* from the same family, were significantly associated with markers of immune activation. Members of family *Lachnospiraceae* have been shown to degrade mucins, potentially increasing gut permeability. Also, genera *Dorea* and *L-Ruminococcus* colonizing mucin regions of the GI tract can degrade mucins and metabolize the released sialic acids (57–59). Negative correlation of plasma IPA and IAA with this family could be illustrative of the relationship between mucin-associated bacteria that increase gut permeability and thus potentially induce immune activity. For example, *Dorea* was positively associated with T-cell production of IFN-γ and *L-Ruminococcus* with LPS-stimulated production of IL-6 (**Table 2**). This finding for *Dorea* is confirmed by a previous study which also showed that higher abundance of *Dorea* is associated with increased production of IFN-γ (60), though the association of *L-Ruminococcus* with IL-6 production is apparently novel. The negative association of *Lachnospiraceae* with effector-memory CD4 T-cells expressing the activation marker CD38 is not obviously consistent with the immune activation associated with this group of bacteria at the family level. It is likely that these effects are genus-specific. In summary, these data tend to support the suggestion that some *Lachnospiraceae* family member may cause immune stimulation, but the picture is complicated and further work involving experimental approaches in model systems is needed to confirm the association of *L-Ruminococcus* with IL-6 production.

In summary, our examination of the association of Trp metabolites with a broad spectrum of 88 immune markers showed that the Kyn/Trp ratio, a marker of IDO activity, was strongly associated with several markers of systemic inflammation, showing ongoing immune activation in a group of healthy adults without active acute or chronic disease. The Kyn/Trp ratio was positively associated with age and was higher in obese compared to non-obese individuals, emphasizing the association of these two factors with immune activation. In addition, commensal bacteria negatively associated with plasma indole metabolite concentrations were also associated with markers of systemic immune activation, suggesting that these taxa, perhaps by decreasing the availability of Trp metabolites such as indole, IAA and IPA to host tissues, increase the risk of systemic inflammation triggered by disruption of the mucosal barrier by these bacteria. Another possibility is that the relative abundance of these microbial taxa could be higher in a slightly more inflamed environment associated with lower levels of Trp metabolites for a yet to be identified another reason.

## Data availability statement

The 16S rRNA sequence data presented in the study are deposited in the European Nucleotide Archive, (https://www.ebi.ac.uk/ena/browser/home) under accession number PRJEB53463. Further inquiries can be directed to the corresponding author(s).

## Ethics statement

The studies involving human participants were reviewed and approved by University of California Davis IRB. The patients/participants provided their written informed consent to participate in this study.

## Authors contributions

CS, MK and NR jointly conceived the study, designed scientific objectives, and provided editorial and conceptual input to the final version of the manuscript; MK also oversaw 16S rRNA sequence based analysis of microbial community data, analyzed data related to **Figure 5**, and participated in writing; XJ assisted in performing and JN oversaw measurement of plasma tryptophan metabolites and JN also participated in writing; NR participated in writing the statistical analysis plan, performed the statistical analysis, wrote the original manuscript, edited, revised and drafted the manuscript; YA measured immune biomarkers in plasma; XJ, TF and YA all participated in lab work to measure immune variables. All authors read and approved the final manuscript.

## Funding

This research was supported with funding from a USDA Agricultural Research Service (project numbers 2032-51530-026-000-D, 2032-51530-025-00-D, 2032–53000–001-00-D and 2032-51530-022-00-D), as well as the National Center for Advancing Translational Sciences and National Institutes of Health through a grant, UL1 TR001860.

## Acknowledgments

The authors thank Janet Peerson for providing expertise in statistical analysis. We thank Lacey Baldiviez, Eduardo Cervantes, and Yasmine Bouzid for their work with study volunteers. We thank Dr. Ellen Bonnel for her contributions to coordinating and managing the study, including her general oversight of the day-to-day study operations. We thank Leslie Woodhouse and the WHNRC Bioanalytical Support Lab staff including Debra Standridge, Connor Osato and Joe Domek, and the Physiology Support Lab staff, including Justin Waller, for their work on the study.

## Conflict of interest

The authors declare that the research was conducted in the absence of any commercial or financial relationships that could be construed as a potential conflict of interest.

## Publisher’s note

All claims expressed in this article are solely those of the authors and do not necessarily represent those of their affiliated organizations, or those of the publisher, the editors and the reviewers. Any product that may be evaluated in this article, or claim that may be made by its manufacturer, is not guaranteed or endorsed by the publisher.
